# A Randomized Controlled Trial of Metacognitive Therapy for Depression: Analysis of 1-Year Follow-Up

**DOI:** 10.3389/fpsyg.2019.01842

**Published:** 2019-08-08

**Authors:** Odin Hjemdal, Stian Solem, Roger Hagen, Leif Edward Ottesen Kennair, Hans M. Nordahl, Adrian Wells

**Affiliations:** ^1^Department of Psychology, Norwegian University of Science and Technology, Trondheim, Norway; ^2^Department of Mental Health, Norwegian University of Science and Technology, Trondheim, Norway; ^3^Nidaros DPS, Division of Psychiatry, St. Olavs Hospital, Trondheim, Norway; ^4^Division of Clinical and Health Psychology, The University of Manchester, Manchester, United Kingdom; ^5^Greater Manchester Mental Health NHS Foundation Trust, Prestwich, United Kingdom

**Keywords:** depression, metacognitive therapy, 1-year follow-up, rumination, worry

## Abstract

This paper reports the 1-year follow-up results from a randomized controlled trial (RCT), which examined the efficacy of metacognitive therapy (MCT) for unipolar depression compared to a waiting condition. Thirty-nine patients with major depression were offered MCT and were divided into two conditions; immediate MCT with 10 weekly sessions or a waiting period that had a 10-week delayed MCT start. Two participants dropped out during the waiting condition. Thirty-four patients participated in the follow-up assessment. Based on the intent-to-treat sample and all patients, 67% were classified as recovered, 13% improved, and 20% were unchanged at 1-year follow-up. For the completers sample 73% recovered, 12% improved, and 15% were unchanged. Five of the 31 patients (13%) that were in remission at post-treatment experienced relapse at 1-year follow-up. Within-group effect sizes were large for reductions in symptoms of depression (*d* = 2.09) and anxiety (*d* = 1.16) at 1-year. Treatment response was associated with reductions in rumination, worry, and metacognitive beliefs as predicted by the metacognitive model, but reductions in metacognitions independently predicted reductions in depression scores from pre-treatment to 1-year follow-up. The results suggest that treatment gains are stable at 1-year follow-up. The study sets the stage for future research, which should evaluate MCT over a longer term and compare it with active treatments using suitably powered RCTs.

## Introduction

Depression is one of the most common psychiatric disorders, with a high degree of comorbidity (Kessler et al., 2003), and is the leading cause of disease burden worldwide (World Health Organization [WHO], 2018). The consequences are significant in terms of lost work productivity, mortality, and lower quality of life (Simon, 2003). The risks associated with depression are profound with the majority of suicides committed by depressed individuals (Hawton et al., 2013).

Perhaps the most challenging aspect of depression with respect to treatment is its recurrent nature. As many as 85% of those that recover from major depressive disorder will have a second episode within 15 years of naturalistic follow-up, and additional episodes will increase the relapse probability by 18% (Mueller et al., 1999). Despite being recognized as a commonly occurring disorder, many patients do not receive the best recommended treatments (Kessler et al., 2008). Furthermore, for those that receive an active treatment, a major problem for depressed patients is the high relapse rate at follow-up (Steinert et al., 2014).

Cognitive-behavioral therapy (CBT) is a recommended treatment for adult unipolar depression (Butler et al., 2006). However, findings suggest that relapse rates are from 29 to 39% within 1 year, and between 40 and 60% within a period of 2 years (Hollon et al., 2006; Vittengl et al., 2007; Dobson et al., 2009). For behavioral activation the 1-year relapse rates were reported as 50%, with continued medication being 53% and medication withdrawal 59% (Dobson et al., 2009). Antidepressant medication has a similar efficacy to CBT in treating depression but relapse rates are between 29 and 60% within one to 2 years (Parker et al., 2008). There is a clear need to develop more effective treatments for depression and to reduce relapse rates after treatment.

Metacognitive therapy (MCT; Wells, 2009) is a treatment that may offer an advance, because it targets specific processes thought to increase risk of depression. It is based on the Self-Regulatory Executive Function model (S-REF; Wells and Matthews, 1994, 1996; Wells, 2000), which proposes that low mood and depression is prolonged by perseverative thinking styles, such as depressive rumination, worry, and other unhelpful self-regulation strategies. This thinking style, called the cognitive attentional syndrome (CAS; Wells and Matthews, 1994) is influenced by positive and negative metacognitive beliefs about uncontrollability and danger of rumination and worry as well as maladaptive executive control of attentional processes.

Empirical studies, such as those of Papageorgiou and Wells (2003) and Solem et al. (2016) have confirmed theoretically consistent relationships between positive metacognitive beliefs, rumination, negative metacognitive beliefs and depression consistent with the model. The model predicts that recovery from depression requires reductions in rumination, worry, and dysfunctional metacognitions, as well as changes in metacognitive beliefs (Wells, 2009). Clarifying the mechanisms of change in MCT may help expand and elaborate understanding of depression and refine the delivery of treatment (Hoffart et al., 2018). Studies should therefore explore if the effects of MCT for depression are related to changes in the hypothesized causal variables.

A meta-analysis of the effects of MCT for anxiety and depression showed that the treatment is effective (Hedges’ *g* = 2.06 compared to wait-list) and potentially more effective than recommended treatments such as CBT at post-treatment (Hedges’ *g* = 0.69 − 0.37) (Normann and Morina, 2018). Across studies of depression, most of which have been small-scale to date, recovery rates for MCT typically range from 66–79% at post-treatment (Wells et al., 2012; Papageorgiou and Wells, 2015; Hjemdal et al., 2017).

A platform trial of treatment-resistant depression with 12 patients found that 66.6% of patients treated with MCT were recovered at post-treatment and 58.3% at follow-up (Wells et al., 2012) using the stringent criteria of Frank et al. (1991). Similarly, a case series by Callesen et al. (2014) reported that three out of four depressed patients were recovered, and a group MCT study by Dammen et al. (2015) found that 91% of 11 patients recovered at 6-months follow-up. At 1-year follow-up, 70% remained recovered, and 80% at 2-year follow-up (Dammen et al., 2016). In 2015, Papageorgiou and Wells also published a trial for group MCT for antidepressant and CBT resistant depression using a baseline-controlled design. The study included 10 patients and showed that 70% were recovered at post-treatment and 6-months follow-up (Papageorgiou and Wells, 2015). An open-trial with 10 comorbid depressed patients also reported 70% recovery rates at 6-month follow-up (Hjemdal et al., 2017). Whilst promising, these trials are small scale and the data must be considered preliminary.

The first RCT included 39 patients with major depression and compared individual MCT with waitlist (Hagen et al., 2017). Results indicated that 79.5% were recovered at post-treatment and 69.2% at 6-months follow-up. Whilst the recovery rates of MCT are very promising, longer follow-up data from randomized trials is required to assess the effects of MCT for depression.

In the present study, we conducted a follow-up analysis of the Hagen et al. (2017) patients 1-year after finishing treatment. Further, we examined the levels of anxiety, rumination, worry, and dysfunctional metacognitions at 1-year follow-up. The study tested whether gains made in these constructs were different at 1-year follow-up in recovered and non-recovered patients.

## Materials and Methods

### Participants

The total sample consisted of 39 participants of which 59% were women (*n* = 23). The mean age was 33.7 years (*SD* = 10.42) ranging from 18 to 54. Three participants were of Asian ethnicity while the remaining were ethnic Norwegian. A total of 41% were single, 38% were married/cohabitants, 13% had partners, and 8% were divorced/separated. With respect to employment, 31% worked full time, 21% had part-time jobs, 21% were students, while 33% received social/welfare benefits. The group had on average 1.08 (*SD* = 1.28) children. Patients who were treated with SSRI were included if they were on a stable dosage and agreed to maintain this dosage throughout the study. However, only three used SSRIs. The majority of participants had been treated previously for their depression (76.9%). With respect to their highest obtained education, 5% had completed elementary school, 44% had completed high school, 13% finished college, and 38% had a master’s degree.

Mean age of onset for the first depressive episode was 26.2 years (*SD* = 11.7) and patients had suffered from depression on average for 7.6 years (*SD* = 7.1). In the study 84.6% (33 patients) were diagnosed with recurrent depression (one mild, 21 moderate, 11 severe), and 15.4% (six patients) with single depressive episode (three moderate, three severe). Comorbidity was common as only 33% had depression as their single diagnosis. Different additional axis-I disorders were present in 41% of the sample (10 with generalized anxiety disorder, two with panic disorder, and single incidents of social phobia, hypochondriasis, trichotillomania, and eating disorder not otherwise specified). With respect to axis-II disorders, 33% were diagnosed with such (three with avoidant personality disorder and 10 with obsessive compulsive personality disorder). Only three reported having received psychological treatment between post-treatment and 1-year follow-up, 28 reported no additional treatment and eight had missing data on this issue.

### Procedure

The RCT was registered at ClinicalTrials.gov (NCT01608399). The Regional Medical Ethics Committee in Norway (REK-Midt ref. no. 2011/1138) provided their ethical approval. The main inclusion criterion was a DSM-IV diagnosis of primary unipolar depression (including mild, moderate, and major). Participants with a single episode of depression or recurrent depression were included. Further inclusion criteria incorporated signing the written informed consent form and being 18 years or older, accepting random allocation, and not receive multiple therapies at the same time. Patients were excluded if they suffered from a known somatic disease, were psychotic, suicidal, had PTSD, cluster A or cluster B personality disorder, substance dependence, and they had to accept random allocation, and not receive multiple therapies at the same time. In all, 105 participants attended a diagnostic interview of which 63% (*n* = 66) of those were excluded. Reasons for exclusions among were: other primary diagnosis (30%), GAD as the prominent diagnosis (27%), cluster A or B personality disorder (15%), no psychiatric diagnosis (12%), subclinical depression (8%), somatic disease (3%), PTSD (2%), substance dependence (2%), and psychosis (2%).

**FIGURE 1:**
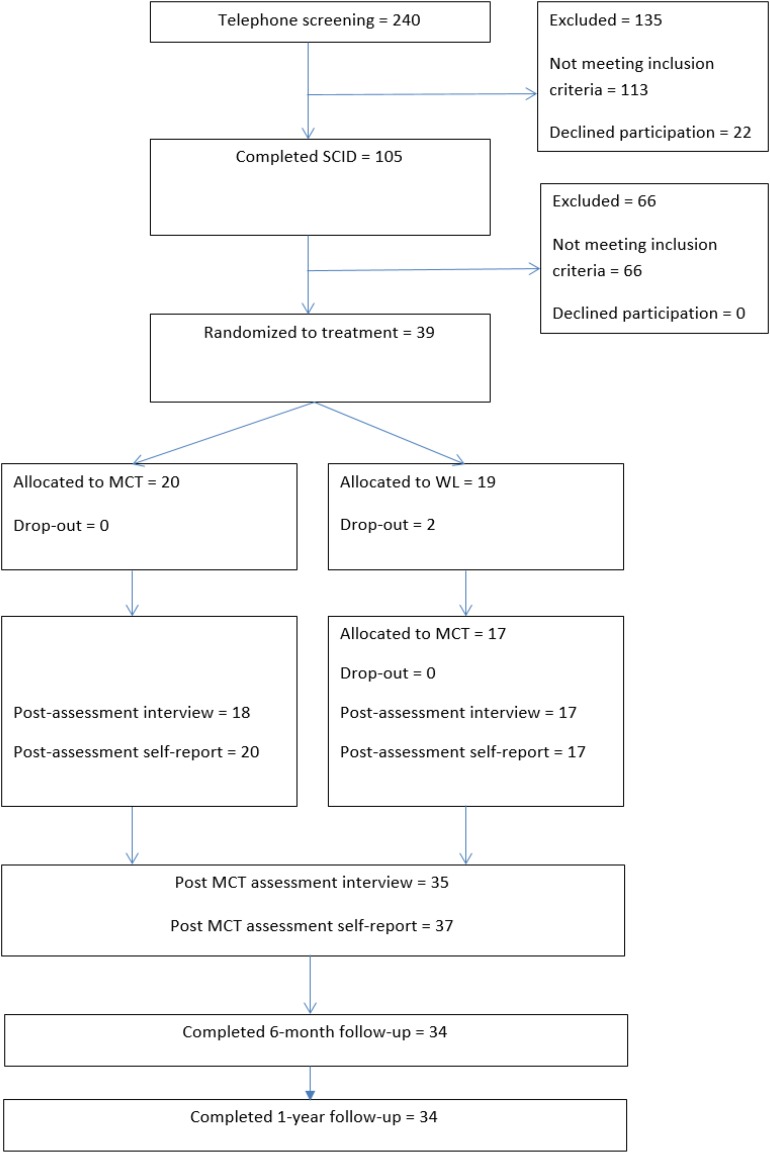
Flow chart.

Participants were recruited between 2013 and 2015. They were treatment-seeking individuals referred by their GP or self-referral. Adverts describing the study were placed in newspapers, in letters to GPs, and on social media. Referred patients were given a telephone screening to ensure that they had symptoms resembling depression. Those that did were offered an appointment to meet with a trained assessor for a diagnostic interview. Further information about the study was given and they were given the informed consent to sign. The assessment covered inclusion and exclusion criteria. The diagnostic interviews used the Structured Clinical Interview for the DSM-IV axis-I (SCID-I; First et al., 1995), as well as the Structured Clinical Interview for the DSM-IV axis-II (SCID-II; Gibbon et al., 1997). The assessment team interviewed patients at pre- and post-treatment. Those accepted into the trial were randomly assigned to begin 10 sessions of MCT treatment either immediately or after a 10-week wait period. Follow-up assessment (1-year) was accomplished by mailing paper versions of the questionnaires to participants, who filled them out in their own homes. Results from the two treatment conditions (immediate and delayed) were included in the analyses of the follow-up data. Figure 1 displays a flow chart of the study.

### Instruments

The Beck Depression Inventory (BDI; Beck et al., 1961) assesses severity of depressive symptoms. The BDI has 21-items that are rated on a 0–3 scale. The reported Cronbach’s alpha of BDI is between 0.86 and 0.89 (Beck et al., 1961, 1988). The BDI is a reliable and valid measure of depressive symptoms (Beck et al., 1988). BDI total scores can be classified accordingly: 0–9 minimal depressive symptoms, 10–18 mild depressive symptoms, 19–29 moderate depressive symptoms, and 30–63 severe depressive symptoms.

The Ruminative Response Scale (RRS; Nolen-Hoeksema and Morrow, 1991) assesses rumination in response to depressed mood (e.g., think “Why do I have problems other people don’t have?” or “think about how sad you feel”). The RRS has 22 items that are rated on a 1 to 4 scale, and scores range from 22 to 88. Higher scores indicate higher levels of rumination. Psychometric properties with Cronbach’s alphas have been reported between 0.88 and 0.92 (Luminet, 2004).

The Positive Beliefs about Rumination Scale (PBRS; Papageorgiou and Wells, 2001b) assesses beliefs about the benefits of rumination (e.g., “Ruminating about my feelings helps me to recognize the triggers for my depression” and “I need to ruminate about the bad things that have happened in the past to make sense of them”). The PBRS has nine items using a 1–4 scale, and scores range from 9 to 36. Good psychometric properties have been documented with Cronbach’s alpha of 0.89 (Luminet, 2004).

The Negative Beliefs about Rumination Scale (NBRS; Papageorgiou and Wells, 2001a) assesses beliefs about uncontrollability and harm as well as interpersonal consequences (e.g., “rumination can make me physically ill,” “I can’t stop myself from ruminating,” “only weak people ruminate”). The NBRS has 13 items using a 1 to 4 response scale, and scores range from 12 to 52. Good psychometric properties have been documented with Cronbach’s alphas between 0.80 and 0.83 (Luminet, 2004).

Metacognitions Questionnaire-30 (MCQ-30; Wells and Cartwright-Hatton, 2004) assesses levels of metacognitive beliefs. The MCQ-30 has 30 items which are rated 1–4, with higher scores indicating higher levels of maladaptive metacognitions. Scores range from 30 to 120. The psychometric properties are good with Cronbach’s alpha for the total score of 0.88 (Spada et al., 2008).

Penn State Worry Questionnaire (PSWQ; Meyer et al., 1990) assesses levels of worry. The PSWQ has 16 items which are rated on a 1–5 scale, with higher scores indicating higher levels of worry. Scores range from 16 to 80. The psychometric properties are good with Cronbach’s alpha of 0.93 (Brown et al., 1992).

The Beck Anxiety Inventory (BDI; Beck and Steer, 1990) assesses severity of anxiety symptoms. The BAI has 21 items which are rated on a 0 to 3 scale. BAI total scores can be classified accordingly: 0–7 minimal anxiety, 8–15 mild anxiety, 16–25 moderate anxiety, and 26–63 severe anxiety. The BAI has good psychometric properties with Cronbach’s alpha of 0.92 (Steer et al., 1993).

### Therapists

Four therapists all of whom were clinical psychologists and were trained in MCT delivered therapy. Treatment was supervised by the last author (AW) and the supervision was based on videotaped recordings of the sessions. In addition, the therapists met every second week for peer supervision.

### Treatment

Treatment consisted of 10 sessions and followed the manual of MCT for depression (Wells, 2009). The main components of the treatment involve in the following sequence; (1) case conceptualization and (2) socialization to the MCT model for depression, (3) learning triggers for rumination, (4) attention training, (5) challenging beliefs about uncontrollability of rumination, (6) challenging other negative metacognitive beliefs, (7) challenging positive metacognitive beliefs, (8) eliminating coping strategies, and (9) relapse prevention.

### Statistics

Shapiro-Wilk test of normality was not significant for the study outcome variable. Effect sizes were calculated with Cohen’s *d*. To evaluate clinically significant outcomes, the corrected Jacobson criterion (Jacobson et al., 1999) reported in Christensen and Mendoza (1986) was used. This meant a cut-off of 14 points and below on the BDI and based on the current sample an estimated reliable change index of 9.49, which was rounded down to 9.

Bivariate Pearson’s correlations were run in order to examine the association between change score from pre-treatment to 1-year follow-up ΔBDI score, and pre-treatment as well as change scores from pre-treatment to 1-year follow-up on: ΔBAI, ΔRRS, ΔNBRS, ΔPBRS, ΔPSWQ, and ΔMCQ-30.

A multiple hierarchical regression analysis explored if changes from pre-treatment to 1-year follow-up in rumination, worry or metacognition predicted change in depressive symptoms from pre-treatment to 1-year follow-up, thus the outcome variable was change from pre-treatment to 1-year follow-up ΔBDI score. In the first step gender and age was entered, in the second step using the forward selection method change scores from pre-treatment to 1-year follow-up were entered for ΔRRS, ΔPSWQ and ΔMCQ-30. Note that pre-treatment scores for the waiting list patients were assessed post-waiting list before starting treatment.

### Missing Values and Imputation of Data

Missing data in the intention to treat (ITT) analyses were replaced using last observation carried forward. Two participants allocated to waiting list (delayed treatment) dropped out during the waiting period (one moved and one started treatment at a private practice psychologist) and did not provide data after pre-treatment. A further two from the waitlist condition did not complete all 10 treatment sessions. In the MCT immediate treatment group all participants completed treatment. All except one of the remaining participants completed self-report questionnaires at 6-month and 1-year follow-up. There was very little missing data on individual BDI items (0.4%) and BAI items (0.8%).

## Results

The results displayed in Table 1 show a significant change in BDI, BAI, MCQ-30, NBRS, PBRS, RRS, PSWQ, and MCQ-30 from pre- to post-treatment, 6-month and 1-year follow-up. The uncontrolled effect sizes varied between 2.53 and 1.16. The highest effect sizes were for levels of rumination and depressive symptoms. Table 1 displays mean and standard deviations for all of the outcomes. At 1-year follow-up there was a small but statistically significant increase in BDI symptoms, but the mean score remained low at 8.85 and the effect size was 2.09.

**TABLE 1:** Means, standard deviations at pre-treatment, post-treatment, and 6-month and 1-year follow-up with mixed modeling for the BDI, BAI, MCQ-30, NBRS, PBRS, RRS, PSWQ (*N* = 39).

		***M* (*SD*)**	***Pairwise comparison***	***d***	***F***	**η2p**
BDI	Pre	25.92(7.14)				
	Post	6.64(8.04)	19.28^∗∗∗^	2.53		
	6-month	8.21(9.45)	–1.56	2.11		
	1-year	8.85(9.09)	−2.21^∗^	2.09	88.76^∗∗∗^	0.70
BAI	Pre	20.56(9.23)				
	Post	4.85(7.22)	15.72^∗∗∗^	1.90		
	6-month	7.00(9.57)	–2.15	1.44		
	1-year	9.95(9.03)	–2.10	1.16	48.33^∗∗∗^	0.56
MCQ-30	Pre	66.31(11.82)				
	Post	44.77(11.81)	0.71^∗∗∗^	1.82		
	6-month	45.15(12.31)	0.00	1.75		
	1-year	45.10(12.51)	0.01	1.74	59.73^∗∗∗^	0.61
NBRS	Pre	27.74(6.05)				
	Post	18.74(5.65)	8.95^∗∗∗^	1.54		
	6-month	18.56(5.42)	0.40	1.59		
	1-year	18.33(5.64)	0.45	1.61	51.80^∗∗∗^	0.58
PBRS	Pre	19.61(6.52)				
	Post	11.82(4.98)	8.24^∗∗∗^	1.34		
	6-month	12.26(4.91)	–0.92	1.27		
	1-year	12.36(5.21)	–1.03	1.23	43.50^∗∗∗^	0.55
RRS	Pre	57.33(6.74)				
	Post	32.97(12.38)	24.45^∗∗∗^	2.44		
	6-month	34.13(12.65)	–1.03	2.29		
	1-year	34.51(12.88)	–1.03	2.22	93.09^∗∗∗^	0.72
PSWQ	Pre	56.36(10.61)				
	Post	39.28(11.02)	16.89^∗∗∗^	1.62		
	6-month	40.61(12.36)	–1.16	1.45		
	1-year	41.05(11.97)	–1.97	1.43	34.15^∗∗∗^	0.49

*BDI, Beck Depression Inventory; BAI, Beck Anxiety Inventory; MCQ-30, Metacognitions Questionnaire-30; PSWQ, Penn State Worry Questionnaire; NBRS, Negative Beliefs about Rumination Scale; PBRS, Positive Beliefs about Rumination Scale; RRS, Ruminative Response Scale. The reported data are based on intention-to-treat. Missing data is replaced using last observation carried forward ^∗∗∗^*p* < 0.001.*

### Clinically Significant Change Analyses

On the BDI, based on Jacobson criteria (Jacobson et al., 1999) at 1-year follow-up the response rates are presented in Table 2. Statistics for ITT and completer samples are presented for the entire combined samples (immediate MCT plus delayed MCT) and for the immediate MCT subgroup seperately. The proportion of recovered patients is higher in the completers data-set compared to the ITT data-set as might be expected. We will concentrate on the ITT data here as it is more conservative since we would expect depressed patients to recover over time and therefore using LOCF is likely to reduce the time effect. Seventy per-cent (70%) of the immediate MCT patients were recovered at 1 year follow-up (*n* = 20), whilst this figure was 66.7% in the combined sample (*n* = 39). A further 15 and 12% of patients were reliably improved and some patients were classified as no change (15 and 20.5%) respectively in the two sub-groupings.

**TABLE 2:** Clinically significant change in depressive symptoms for the MCT immediate treatment group (*n* = 20) and the total combined sample (*N* = 39).

		**Pre-treatment to 1-year follow-up**		
	***N***	**No change**	**Improved**	**Recovered**
**BDI**				
MCT ITT	20	15.0%	15.0%	70.0%
MCT completers	18	16.6%	5.6%	77.8%
All ITT	39	20.5%	12.8%	66.7%
All completers	34	14.7%	11.8%	73.5%

*MCT ITT, MCT immediate treatment group with intention to treat; completers, analysis of those completing all data; All, the total sample (immediate MCT plus delayed MCT); ITT, Intention-to-treat; BDI, Beck Depression Inventory; No patients deteriorated; BDI criteria for improvement, patients that had a 9-point reduction on the BDI or BDI of 14 or less; BDI criteria for recovery, patients that had a 9-point reduction on the BDI and a score of 14 points or lower.*

To check if the recovery rates for the entire sample depended on severity of depression, clinically significant change was also calculated separately for the subgroups *moderate* and *severe depression*. For the *moderate depression* subgroup (*n* = 24), 60% were classified as recovered at 1-year follow-up (72% improved). For the *severe depression* subgroup (*n* = 14) 79% were recovered at 1-year follow-up (93% improved).

Table 3 presents the clinically significant change score from post-treatment to 1-year follow-up. None of the patients that were classified as unchanged at post-treatment had changed their status at 1-year follow-up. Of the improved category one out of five patients changed their classification to recovered. Among the recovered group at post-treatment, 25 patients remained in the recovered category, while one changed to the improved group. Five patients that were recovered at post-treatment changed to unchanged at 1-year follow-up (indicating a relapse rate of 12.8%). The overall picture is that the large majority of recovered patients remained the same both at post-treatment and at 1-year follow-up. The fluctuation is relatively limited, and the data are based on intention-to-treat which is conservative in this regard. The results suggest a small proportion of patients relapsing following MCT at 1-year follow-up.

**TABLE 3:** Change in clinical improvement rates from post-treatment to 1-year follow-up (*N* = 39).

		**1-year follow-up**			
		**Unchanged**	**Improved**	**Recovered**	**Total**
Post-treatment	Recovered	5	1	25	31
	Improved	0	4	1	5
	Unchanged		0	0	3
Total		8	5	26	39

*BDI criteria for improvement, patients that had a 9-point reduction on the BDI. BDI criteria for recovery, patients that had a 9-point reduction on the BDI *and* a score of 14 points or lower.*

To explore any effect of pre-treatment symptom severity on longer term outcomes pre-treatment scores on the BDI, BAI, RRS, PSWQ, NBRS, PBRS, MCQ-30 and BAI were correlated with changes from pre-treatment to 1 year follow up on BDI. None of the pre-treatment scores correlated with the ΔBDI.

Next, we explored the possible association between change in these predictors over the longer-term (pre-treatment to 1-year follow-up) and longer-term change in depression (pre-treatment to 1-year follow-up). The results of these analyses are displayed in Table 4. It is evident that changes in all variables were positively associated with change in depression. The highest correlation was BAI which probably reflects the overlap of symptoms of anxiety and depression. Of the theoretical predictors (purported causal factors) metacognitive belief change (MCQ30) showed the strongest positive association.

**TABLE 4:** Bivariate Pearson’s correlations between the BDI 1-year follow-up score and the change scores from pre-treatment to 1-year follow-up of BDI, BAI, RRS, PSWQ, NBRS, PBRS, and MCQ.

	**1**	**2**	**3**	**4**	**5**	**6**	**7**
1. BDI Δ							
2. BAI Δ	0.69^∗∗^						
3. RRS Δ	0.45^∗∗^	0.46^∗∗^					
4. PSWQ Δ	0.34^∗^	0.31	0.61^∗∗^				
5. MCQ-30 Δ	0.62^∗∗^	0.59^∗∗^	0.65^∗∗^	0.58^∗∗^			
6. NBRS Δ	0.45^∗∗^	0.33^∗^	0.54^∗∗^	0.35^∗^	0.64^∗∗^		
7. PBRS Δ	0.48^∗∗^	0.34^∗^	0.49^∗∗^	0.58^∗∗^	0.63^∗∗^	0.42^∗∗^	

Δ, *changes from pre-treatment to 1-year follow-up scores; BDI, Beck Depression Inventory; BAI, Beck Anxiety Inventory; MCQ-30, Metacognitions Questionnaire-30; PSWQ, Penn State Worry Questionnaire; NBRS, Negative Beliefs about Rumination Scale; PBRS, Positive Beliefs about Rumination Scale; RRS, Ruminative Response Scale. *^∗^ p* < 0.05, ^∗∗^*p* < 0.01, ^∗∗∗^*p* < 0.001.*

Finally, we ran a hierarchical multiple linear regression to explore the best independent predictor amongst change in the predictive mechanisms (MCQ30, RRS, PSWQ). In the first step we controlled for age and gender and neither were significant, in the second step the forward method resulted in ΔMCQ-30 as a significant predictor. Neither ΔRRS nor ΔPSWQ were significant predictors. The summary statistics are presented in Table 5.

**TABLE 5:** A hierarchical multiple regression analysis with changes from pre-treatment to 1-year follow-up ΔBDI scores as dependent variable.

	**Variables**	**Fcha**	***r*^2^cha**	**β**	**Partial *r***	***t*-value**	**Significant**
Step 1 enter		2.20	0.12				
	Age			−0.09		−0.51	n.s.
	Gender			0.33		1.98	n.s.
Step 2 forward		16.53	0.31				
	ΔMCQ-30			0.58		4.07	0.000
	ΔRRS			0.09	0.10	0.53	n.s.
	ΔPSWQ			−0.08	−0.09	−0.49	n.s.

*Predictors are age, gender, change scores from pre-treatment to 1-year follow-up* Δ*RRS,* Δ*PSWQ and* Δ*MCQ-30 using forward variable selection.* Δ, *changes from pre-treatment to 1-year follow-up scores; BDI, Beck Depression Inventory; MCQ-30, Metacognitions Questionnaire-30; PSWQ, Penn State Worry Questionnaire; RRS, Ruminative Response Scale.*

## Discussion

The follow-up results from this RCT showed that the effects gained with MCT at post-treatment were largely maintained at 1-year follow-up for depressive and also for anxiety symptoms. The clinically significance analyses showed that 70.0% of the MCT immediately treated intent-to-treat sample, and 73.5% in the completers sample achieved recovery at 1-year follow up for individual MCT. For the total ITT sample this figure was 66.7%. These results appear to be consistent with previous studies with 1-year follow-up of group MCT depression in an open trial (Dammen et al., 2016) and for individual MCT reported in a previous platform trial for treatment resistant depression (Wells et al., 2012). The current study extends previous findings by including a larger sample size and a randomized controlled design. Results suggest that the majority of patients benefitted from MCT.

One of the major challenges with depression treatment has been the recurring nature of the disorder. Relapse rates for CBT have been reported as 29–39% at one year and up to 60% for antidepressant treatments with a range between 40 to 60% within 2 years (Gloaguen et al., 1998; Hollon et al., 2006; Vittengl et al., 2007; Dobson et al., 2009). In the current study five of the patients who were recovered at post-treatment relapsed which is a rate of 12.8%, and in addition one was classified as improved instead of recovered at follow-up. Of the five patients who were improved at post-treatment, four remained improved at follow-up, while one had improved further and classified as recovered. The beneficial effects of MCT seem to cut across the severity of symptoms with 60% recovered in the *moderate depression* subgroup and 79% recovered in the *severe depression* subgroup. Also, there were no pre-treatment (*t* = 0.54, *p* = n.s.) nor post-treatment (*t* = 1.09, *p* = n.s.) differences in level of depressive symptoms in the current sample between patients with and without personality disorder. This suggests that the treatment effects may not be dependent on the presence or absence of at least some co-morbid personality issues.

Some exploratory results from the present paper showed that the pre-treatment values were not associated with the 1-year follow-up values of depression while the change scores from pre-treatment to 1-year follow-up were. This suggests that pre-treatment severity was not associated with depression improvement levels over the 1-year period. The results of bivariate correlations showed that change in patients’ metacognitive beliefs, rumination and worry over the 1-year rather than pre-scores on depression symptom severity were associated with changes from pre-treatment to 1-year follow-up depression scores. Consistent with the Metacognitive model (Wells, 2009), reduction in rumination, worry and maladaptive metacognitions appeared to be associated with improvements in depression over the longer term. Among these processes, the regression showed that changes in maladaptive metacognitions was an independent predictor of changes in depression scores from pre-treatment to 1-year follow-up. Similar findings have been found in studies of predictors of outcome in OCD treatment (Solem et al., 2009). Future studies should explore metacognitions measured session by session, which will highlight the possibility to disaggregate both the within and between effects.

Metacognitive therapy could be more effective than other treatments (e.g., Nordahl et al., 2018; Normann and Morina, 2018) and could have good long terms outcomes. There are different explanations as to why MCT might offer an efficient and long-lasting treatment for depression. MCT for depression aims to: (1) increase awareness of metacognitive processes and reduce of rumination, worry and threat monitoring; (2) facilitate control of these processes and greater attentional flexibility, and (3) modify negative and positive metacognitive beliefs (Wells, 2009). The S-REF model (Wells and Matthews, 1994), which is the founding of MCT, hypothesizes that the cognitive attentional syndrome maintains disorder and MCT is designed to directly target this mechanism. In the present study those who recovered had a considerably larger reduction in rumination, negative and positive beliefs about rumination, negative metacognitions, and worry, than patients who did not recover. Furthermore, this mechanism is thought to underlie most forms of psychopathology and so MCT may be particularly effective at dealing with multiple morbidities, thereby reducing parallel problems that may confer risk of relapse (e.g., Nordahl, 2009; Hjemdal et al., 2017). The present findings are consistent with the metacognitive theory, and are in line with other studies showing that metacognitions and rumination are important factors for the level of symptoms of depression (Papageorgiou and Wells, 2003; Wells, 2009; Solem et al., 2016). The research is also in line with research showing that change in metacognition is associated with change in symptoms (Solem et al., 2009).

### Limitations

One of the limitations of the study is the small sample size. In addition, five patients (12.8%) did not attend the follow-up assessment, and two of these dropped out early when they were randomized to the waitlist condition. We used last observation carried forward to deal with these missing data. This method has been criticized, but depression is known to recover over time, and we retained the last scores of patients that dropped out which reduces this effect of time. Another limitation is that follow-up assessment was based on self-reported data. Future studies should include additional diagnostic evaluations and compare MCT to other active treatment.

The limitations reported in Hagen et al. (2017) are also valid for the current study. There was only informal assessment of treatment adherence and therapist competence. Adherence was, monitored through supervision but there was no formal assessment of adherence to the treatment manual. As previously reported, there were no differences between therapists in terms of patient outcomes. This suggests that therapist differences did not affect the results. Future studies should include active treatment as a comparison condition. However, the course of untreated depression may serve as a benchmark for assessing the true benefits of an active treatment. Posternak and Miller (2001) reported that the decrease in depressive symptomatology can be between 10 and 15% on average without treatment.

The sample included cluster C personality disorders which applied for 33% of the sample, but other personality disorders were not included. The results are therefore limited to cluster C personality disorder and predominantly OCPD and avoidant personality disorders.

## Conclusion

Large improvements in depression and anxiety symptoms were observed. Improvement was associated with reductions in rumination, worry and metacognitions. The treatment gains were sustained at 1-year follow-up. Improvement in metacognitive beliefs (a hypothesized mechanism) showed a unique positive association with improvement in depression symptoms over 1 year. The current low relapse rates (12.8%) indicate that MCT is a potentially effective treatment for depression, but further studies comparing MCT for depression with other treatments are needed.

## Data Availability

The raw data supporting the conclusions of this manuscript will be made available by the authors, without undue reservation, to any qualified researcher.

## Ethics Statement

All subjects gave a written informed consent in accordance with the Declaration of Helsinki. The trial was registered at ClinicalTrials.gov and approved by the Regional Medical Ethics Committee in Norway (ref. no. 2011/1138).

## Author Contributions

RH, OH, SS, and LK conducted the therapy in the trial. AW supervised the therapists. All authors have contributed in the writing of the manuscript.

## Conflict of Interest Statement

The authors declare that the research was conducted in the absence of any commercial or financial relationships that could be construed as a potential conflict of interest. The reviewer SJ declared a past collaboration with the authors HN and AW to the handling Editor.
